# Knowledge, attitudes, and practices among physicians and pharmacists toward antibiotic use in sepsis

**DOI:** 10.3389/fmed.2024.1454521

**Published:** 2025-01-15

**Authors:** Jingmin Zhang, Haipeng Shi, Yanmei Xia, Zhenghua Zhu, Yaojun Zhang

**Affiliations:** ^1^Department of Intensive Care Unit, Shanxi Bethune Hospital, Shanxi Academy of Medical Sciences, Tongji Shanxi Hospital, Third Hospital of Shanxi Medical University, Taiyuan, Shanxi, China; ^2^Department of Intensive Care Unit, Tongji Hospital, Tongji Medical College, Huazhong University of Science and Technology, Wuhan, Hubei, China; ^3^Heping Hospital Affiliated to Changzhi Medical College, Changzhi, Shanxi, China; ^4^Linfen Central Hospital, Linfen, Shanxi, China

**Keywords:** knowledge, attitudes, practices, antibiotic, sepsis, cross-sectional study

## Abstract

**Background:**

Sepsis management in the Intensive Care Unit (ICU) presents a significant challenge within contemporary healthcare. The primary challenge lies in ensuring the timely and appropriate utilization of antibiotics. Inappropriate antibiotic use in sepsis management can result in a multitude of adverse outcomes. There has been insufficient focus on thoroughly understanding and resolving the issues related to the improper application of antibiotics in sepsis treatment by physicians and pharmacists. This gap in research is concerning, considering its potential implications for patient outcomes and public health. This study aimed to assess the knowledge, attitudes and practices (KAP) among physicians and pharmacists toward antibiotic use in sepsis.

**Methods:**

This web-based cross-sectional study was conducted at Shanxi Bethune Hospital between June 2023 and October 2023. A self-designed questionnaire was developed to collect demographic information of physicians and pharmacists, and to assess their knowledge, attitudes and practices toward antibiotic use in sepsis.

**Results:**

A total of 200 valid questionnaires were collected. Among the participants, 115 (57.5%) were female and 118 (59%) had experience with ICU patient management. The mean knowledge, attitudes and practices scores were 10.2 ± 1.14 (possible range: 0–12), 45.88 ± 4.00 (possible range: 10–50) and 48.38 ± 5.84 (possible range: 11–55), respectively. Multivariate logistic regression showed that attitudes (OR = 1.59, 95%CI: 1.34–1.87, *p* < 0.001), work experience of 15 years and above (OR = 7.17, 95%CI: 2.33–22.0, *p* = 0.001) were independently associated with proactive practices. For physicians, the structural equation model (SEM) demonstrated that attitudes directly affects practices, as indicated by a path coefficient of 0.91 (*p* < 0.001). For pharmacist, SEM showed that knowledge directly affect attitudes, with a path coefficient of 0.75 (*p* < 0.024), moreover, attitudes directly affect practices, with a path coefficient of 0.87 (*p* < 0.001).

**Conclusion:**

The findings revealed that physicians and pharmacists have sufficient knowledge, active attitudes, and proactive practices toward the antibiotic use in sepsis. Nonetheless, the findings also reveal the persistence of certain misconceptions, alongside notable shortcomings in both attitudes and practices. Comprehensive training programs are imperative for enhancing the practices of physicians and pharmacists in this field.

## Introduction

Sepsis in the intensive care unit (ICU) presents a significant challenge in modern healthcare (1). The mortality associated with sepsis varies from 15% in patients with sepsis without shock to 56% in those with sepsis accompanied by shock (2). The primary pathogens associated with sepsis include *Pseudomonas aeruginosa*, *Staphylococcus aureus*, and *Candida albicans*, all of which exhibit serious multi-drug resistance (3), and the management of sepsis in the ICU is heavily reliant on the use of antibiotics (4).

In recent years, sepsis management has emphasized early identification and prompt antibiotic administration as key strategies to improve outcomes. Guidelines now recommend initiating antibiotics within 1 h for patients with septic shock or a high likelihood of sepsis, as early antibiotic use has been linked to reduced mortality, particularly in septic shock cases (5). To optimize treatment, the choice of antibiotics should consider patient history, comorbidities, and local resistance patterns, with adjustments as culture results become available (6).

Challenge lies in the timely and appropriate use of these antibiotics. Inappropriate use of antibiotics in sepsis management can lead to several adverse outcomes. Previous research highlights that these adverse effects encompass detrimental drug interactions, escalated toxicity, the emergence of antibiotic resistance, and the liberation of endotoxins, which are crucial in the pathogenesis of sepsis and septic shock (7, 8). Subsequent studies have established a correlation between the inappropriate administration of antibiotics and an increase in both mortality and adverse clinical events (9). Moreover, the emergence of multi-drug resistant strains complicates the choice of empirical antibiotic therapy, making it crucial to consider local microbial and resistance patterns (10).

Despite the indispensable role antibiotics play in sepsis management, a noteworthy gap persists in the current research landscape. Inadequate attention has been devoted to a comprehensive understanding and resolution of the inappropriate application of antibiotics in the treatment of sepsis by medical practitioners and pharmacists. This research deficit raises significant concerns, given the potential repercussions for patient outcomes and public health (11, 12). Consequently, a meticulous examination of the knowledge and attitudes of these professional groups regarding antibiotic utilization in sepsis becomes imperative to cultivate judicious clinical practices. While numerous studies have explored antibiotic perceptions, none are specific to this critical area of sepsis management (13–15).

The conceptual framework of knowledge, attitudes, and practices (KAP) research serves as a valuable lens for exploring the intricate dynamics surrounding antibiotic utilization in sepsis (16, 17). Thus, this study aimed to address the current knowledge gap by elucidating the complexities of attitudes and identifying the actual practices influencing antibiotic usage in the critical context of sepsis. This study aims to support the development of targeted clinical interventions.

## Materials and methods

### Study design and participants

This cross-sectional survey was conducted at Shanxi Bethune Hospital between June 2023 and October 2023. Participants in the study comprised physicians and clinical pharmacists affiliated with this institution, with a total of 200 valid responses collected. In the study population, 127 participants were classified as physicians. In the context of the Chinese medical system, fully licensed physicians may hold bachelor’s or master’s degrees in medicine. No nurse practitioners, physician assistants, or other roles were included in this study. This classification aligns with the standard definitions of medical practice in China. Notably, only participants in direct patient care roles were included, while other roles, such as nurse practitioners or physician assistants, were excluded. This study was approved by the Ethic Committee of Shanxi Bethune Hospital (No. YXLL-2023-107), which functions as an Institutional Review Board (IRB) at the hospital. Comprehensive review and approval were conducted based on the submitted protocol, including the Application Form for Medical Ethics Review, Sample Informed Consent Form, and Clinical Research Protocol. All participants provided written informed consent prior to participation.

### Questionnaire

The questionnaire was formulated with guidance derived from Sepsis Guidelines (18) and relevant literature on antibiotics management of sepsis (19, 20). The initial draft underwent refinements incorporating feedback from three senior experts, each holding the title of associate professor and specializing in ICU and Clinical Pharmacy. Following these expert revisions, a preliminary trial was conducted on a limited scale (*n* = 41), resulting in a Cronbach’s alpha coefficient value of 0.895, indicating good internal consistency.

The final questionnaire, administered in Chinese, encompassed four dimensions: demographic information, knowledge, attitudes, and practices. Demographic information comprised 11 items, while the knowledge, attitudes, and practices dimensions included 12, 11, and 11 items, respectively. Knowledge items were scored 1 point for a correct answer and 0 points otherwise, resulting in a possible score range of 0–12. The attitude items scored on a five-point Likert scale ranging from very positive (5 points) to very negative (1 point), wherein questions A11 are designated exclusively for descriptive analysis purposes, with a possible score range of 10 to 50. Similarly, practice items were scored on a five-point Likert scale, varying from very consistent (5 points) to very inconsistent (1 point), with a possible score range of 11 to 55 (see Supplementary material).

Data collection was conducted using an online questionnaire hosted on Sojump.^1^ Prior to accessing the questionnaire, participants were required to select the option “I agree to participate in this study,” thereby providing their consent. While no personally identifiable information was collected, a temporary IP restriction was implemented to prevent duplicate submissions. The IP addresses were stored temporarily during the submission process but were not retained after data collection was completed. This approach aligns with ethical practices outlined in previous studies (21, 22).

### Sample size calculation

The sample size calculation was based on a ratio of 5–20 times the number of questionnaire items, as recommended (23). The KAP questionnaire includes 34 items (12 for Knowledge, 11 for Attitude, and 11 for Practice). To account for a potential 10% missing data rate, the target sample size was set to exceed 189 questionnaires.

### Statistical analysis

STATA 17.0 (Stata Corporation, College Station, TX, USA) was employed for statistical analysis. Continuous variables were presented as mean ± SD, while categorical variables were expressed as *n* (%). Continuous variables conforming to a normal distribution were assessed using the t-test or ANOVA. For multivariate analysis, the median (50% distribution) of the total score was used as the threshold value. Pearson correlation was used to analyze the correlation between knowledge, attitudes, and practices. The structural equation model (SEM) of knowledge, attitudes and practices among physicians and pharmacists toward antibiotic use in sepsis was constructed with AMOS 24.0 (IBM, NY, United States). The hypotheses as following: (1) knowledge had direct effects on attitude, (2) knowledge had direct effects on practice, and (3) attitude had direct effects on practice. The model fitting was evaluated with CMIN/DF (Chi-square fit statistics/degree of freedom), RMSEA (root mean square error of approximation), IFI (incremental fix index), TLI (Tucker-Lewis index) and CFI (comparative fix index). Two-sided *p* < 0.05 were considered statistically significant.

## Results

A total of 200 valid questionnaires were collected. Among the participants, 115 (57.5%) were female, 105 (52.5%) aged 36–45 years, 106 (53%) had graduated from college and undergraduate program, 95 (47.50%) had work experience of 6–15 years, 135 (67.5%) in tertiary hospitals, and 118 (59%) had experience in managing ICU patients. Significant demographic differences, including age (*p* < 0.001), marital status (*p* = 0.012), work experience (*p* = 0.022), professional title (*p* = 0.032), and ICU management experience (*p* = 0.015), were observed among participants, which may influence knowledge, attitudes, and practices scores. Therapeutic drug monitoring (TDM) for antibiotics was conducted in hospitals where 45 (22.5%) of the respondents were employed, with sulfonamide antibiotics comprising 46.88% of the TDM. Furthermore, 24 (40%) of the clinical pharmacists were affiliated with the respiratory and general internal medicine departments.

The mean knowledge, attitudes and practices scores were 10.2 ± 1.14 (possible range: 0–12), 45.88 ± 4.001 (possible range: 10–50) and 48.38 ± 5.84 (possible range: 11–55), respectively. The knowledge varied from those with different Professional Title (*p* = 0.032). As for the attitudes, there were difference among physicians and pharmacists with different age (*p* < 0.001), marital status (*p* = 0.012) and work experience (*p* = 0.022). The difference of practices scores were found among physicians and pharmacists with different age (*p* = 0.045) and ICU patient management experience (*p* = 0.015) (Table 1).

**Table 1:** Demographic characteristics and KAP scores.

	*N* (%)	Knowledge		Attitudes		Practice Score	
		Mean ± SD	*p*	Mean ± SD	*p*	Mean ± SD	*p*
*N* = 200		10.2 ± 1.14		45.88 ± 4.001		48.38 ± 5.84	
Gender			0.129		0.415		0.409
Male	85 (42.5)	10.32 ± 1.14		45.6 ± 4.04		48.96 ± 4.99	
Female	115 (57.5)	10.10 ± 1.12		46.08 ± 3.97		47.93 ± 6.38	
Age			0.611		<0.001		0.045
35 years and below	58 (29)	10.12 ± 1.14		47.86 ± 2.79		49.63 ± 5.55	
36–45 years	105 (52.5)	10.23 ± 1.18		45.18 ± 4.14		47.57 ± 5.96	
46 years and above	37 (18.50)	10.21 ± 1.03		44.75 ± 4.17		48.67 ± 5.69	
Residential type			0.605		0.684		0.620
Rural	22 (11)	9.95 ± 1.46		45.54 ± 4.11		48.45 ± 6.70	
Urban	178 (89)	10.23 ± 1.09		45.92 ± 3.99		48.36 ± 5.74	
Marital status			0.638		0.012		0.495
Unmarried/Divorced/Other	25 (12.50)	10.24 ± 1.3		47.64 ± 3.3		49.12 ± 5.70	
Married	175 (87.5)	10.19 ± 1.11		45.62 ± 4.03		48.26 ± 5.87	
Education level			0.541		0.559		0.869
Associate’s and Bachelor’s	106 (53)	10.17 ± 1.14		45.52 ± 4.31		48.21 ± 6.06	
Master’s	86 (43)	10.24 ± 1.16		46.17 ± 3.60		48.44 ± 5.59	
Doctorate	8 (4)	10 ± 0.75		47.37 ± 3.62		49.75 ± 6.04	
Work experience			0.197		0.022		0.268
Less than 5 years	47 (23.50)	9.83 ± 1.47		46.12 ± 4.03		47 ± 6.46	
6–15 years	95 (47.50)	10.32 ± 0.96		46.55 ± 3.58		48.82 ± 5.39	
15 years and above	58 (29)	10.29 ± 1.04		44.56 ± 4.36		48.75 ± 5.95	
Hospital level			0.731		0.859		0.962
Tertiary Hospital	135 (67.5)	10.18 ± 1.24		46.02 ± 3.82		48.28 ± 5.93	
Other	65 (32.5)	10.23 ± 0.89		45.58 ± 4.35		48.56 ± 5.68	
Current position			0.826		0.109		0.140
Intensive Care or Emergency Department Physician	84 (42)	10.29 ± 0.97		46.5 ± 3.69		49.32 ± 5.59	
Infectious Disease Physician	3 (1.5)	10 ± 1		45.66 ± 4.93		50.66 ± 5.85	
Physician in other departments excluding Intensive Care, Emergency, or Infectious Disease	40 (20)	10.32 ± 0.94		44.32 ± 4.62		47.5 ± 5.65	
Clinical Pharmacist	60 (30)	10.05 ± 1.44		45.9 ± 3.70		48.26 ± 5.68	
Hospital Pharmacist	13 (6.5)	9.92 ± 1.11		46.61 ± 4.35		44.92 ± 7.63	
Professional title			0.032		0.168		0.584
Junior	32 (16)	9.84 ± 1.39		46.93 ± 3.81		49.18 ± 5.67	
Intermediate	75 (37.5)	10.16 ± 1.18		45.58 ± 4.04		47.56 ± 6.16	
Associate	75 (37.5)	10.48 ± 0.87		45.94 ± 3.96		48.69 ± 5.74	
Senior	18 (9.00)	9.83 ± 1.20		44.94 ± 4.22		49 ± 5.16	
Experience with ICU patient management			0.392		0.237		0.015
Yes	118 (59)	10.27 ± 1.10		46.25 ± 3.74		49.22 ± 5.52	
No	82 (41)	10.08 ± 1.17		45.34 ± 4.30		47.15 ± 6.10	
Whether hospital’s laboratory offer therapeutic drug monitoring (TDM)			0.623		0.356		0.823
Yes	45 (22.5)	10.26 ± 1.17		46.55 ± 3.50		48.26 ± 5.41	
No	155 (77.5)	10.18 ± 1.13		45.68 ± 4.12		48.40 ± 5.97	

	Frequency	Percentage (%)
Types of antibiotics for which the hospital offers TDM		
Aminoglycoside antibiotics	13	20.31
Penicillin antibiotics	8	12.5
Sulfonamide antibiotics	30	46.88
Glycopeptide antibiotics	3	4.69
Fluoroquinolone antibiotics	10	15.63
Other	13	20.31
If you are a clinical pharmacist, what is your specialization?		
Pediatrics	1	1.67
Other	8	13.33
Respiratory and General Internal Medicine	24	40
Cardiovascular and Anticoagulation	7	11.67
Neurology	5	8.33
Intensive Care Medicine	15	25

Most participants recognized the syndrome’s complexity and the distinction between sepsis and septic shock, with high correctness rates of 94% (K1) and 96.5% (K12), respectively. However, 31.5% of them were still unclear about the timing of using antibiotics to treat sepsis (K7). 25% were unable to clearly recongnize that Enterobacteriaceae bacteria, *Pseudomonas aeruginosa*, or Acinetobacter that express ESBLs are more sensitive to carbapenem antibiotics (K5). Moreover, there was a notable misconception that sepsis is only caused by bacteria, with a correctness rate of only 8% (K3) and that septic shock and sepsis refer to the same disease, with a correctness rate of 3.5% (K2) (Table 2).

**Table 2:** Knowledge.

	Correctness rate *N* (%)
K1. Sepsis is a syndrome in which the body’s response to infection leads to organ dysfunction, characterized mainly by symptoms such as chills, fever (or low body temperature), palpitations, shortness of breath, and changes in mental status.	188 (94)
K2. Septic shock and sepsis refer to the same disease; there is no distinction.	7 (3.5)
K3. Sepsis is solely caused by bacteria; fungi, parasites, and viruses do not cause sepsis.	16 (8)
K4. Risk factors for sepsis include age (young or elderly), compromised immune system, a history of diabetes or cirrhosis, prolonged intensive care unit stays, trauma, invasive procedures (e.g., intravenous catheters or tracheal intubation), and long-term use of corticosteroids, among others.	197 (98.5)
K5. Enterobacteriaceae producing extended-spectrum β-lactamases (ESBL), *Pseudomonas aeruginosa*, or Acinetobacter species are more sensitive to carbapenem antibiotics.	150 (75)
K6. Discontinuing antibiotics as soon as clinical judgment determines that a disease is not sepsis, especially when culture results are negative, is an important measure against antibiotic resistance.	165 (82.5)
K7. Administer antibiotics promptly after confirming sepsis or septic shock; however, there is no recommendation for a specific target time less than 1 h.	137 (68.5)
K8. Depending on the patient’s specific condition, continuous or prolonged administration of *β*-lactam antibiotics may be considered for sepsis patients.	174 (87)
K9. When de-escalating treatment, be cautious not to prolong the total duration of antibiotic administration.	166 (83)
K10. In patients with sepsis and renal dysfunction, adjusting antibiotic dosages may be necessary due to reduced clearance of antibiotics by the kidneys, leading to increased blood drug concentrations.	194 (97)
K11. Tissue distribution concentrations of antimicrobial drugs are influenced by various factors, including drug properties, tissue types, inflammation severity, blood–brain barrier, placental barrier, and others. Therefore, it is necessary to consider these factors comprehensively when using antimicrobial drugs.	199 (99.5)
K12. Septic shock and sepsis are not the same disease; septic shock presents with low blood pressure in addition to the symptoms of sepsis.	193 (96.5)

Attitudes toward antibiotic use in sepsis were generally proactive, with a majority agreeing on the importance of timely knowledge updates, active participation in academic conferences, and collaborative interventions for rational antibiotic use. A significant majority of participants (76.5%) strongly agreed on the importance of timely updating knowledge on sepsis antibiotic guidelines. Moreover, 81.5% of the respondents preferred branded antibiotics when the condition of a sepsis patient worsened significantly, mainly due to perceived better efficacy and safety (Table 3).

**Table 3:** Attitudes.

	Strongly agree	Agree	Neutral	Disagree	Strongly disagree
A1. You consider timely updating knowledge related to sepsis antibiotic guidelines to be highly important.	153 (76.5)	44 (22)	3 (1.5)	/	/
A2. You are enthusiastic about actively participating in academic conferences related to sepsis and rational antibiotic use, and exchanging clinical experiences about the appropriate use of antibiotics for sepsis with colleagues.	140 (70)	54 (27)	6 (3)	/	/
A3. As a pharmacist/physician, you are willing to collaborate with clinical departments/pharmacy departments to discuss interventions related to the rational use of antibiotics for sepsis, dose adjustments, blood drug concentration monitoring, and other aspects.	150 (75)	46 (23)	4 (2)	/	/
A4. During the use of antibiotics, you give importance to patients’ biochemical indicators such as PCT, CRP, and microbial culture results to guide the anti-infection treatment plan.	147 (73.5)	52 (26)	1 (0.5)	/	/
A5. You recognize that the overuse of antibiotics can lead to the emergence of drug-resistant bacteria.	154 (77)	45 (22.5)	1 (0.5)	/	/
A6. If antibiotic treatment plans prove to be ineffective, you are willing to proactively communicate with your clinical team and clinical pharmacists to discuss alternative treatment approaches.	145 (72.5)	53 (26.5)	2 (1)	/	/
A7. You maintain a cautious attitude toward off-label drug use.	84 (42)	98 (49)	15 (7.5)	2 (1)	1 (0.5)
A8. You acknowledge the importance of scientifically implementing de-escalation antibiotic strategies for sepsis.	117 (58.5)	79 (39.5)	3 (1.5)	1 (0.5)	/
A9. You believe that you have a good grasp of adjusting antibiotic dosages based on liver and kidney function for sepsis patients.	58 (29)	94 (47)	44 (22)	4 (2)	/
A10. You recognize the significance of antibiotic blood concentration monitoring in the treatment of sepsis patients.	118 (59)	74 (37)	8 (4)	/	/

	a. Branded Antibiotics	b. Generic Antibiotics	c. Both are acceptable
A11. When the condition of a sepsis patient worsens significantly, do you prioritize the use of branded antibiotics or generic antibiotics?	163 (81.5)	3 (1.5)	34 (17)
A11.1 If you choose branded or generic antibiotics and not the other, what are your reasons? (Multiple choices)			
a. You believe it offers better efficacy and safety compared to the other formulation.	150 (92.02)	0	
b. Considering a cost-effectiveness perspective, you consider it the appropriate choice.	52 (31.9)	3 (100)	
c. Other	2 (1.23)	0	

In practice, a significant number of participants reported regular attendance at relevant academic conferences (39%) and consultation of the latest guidelines (44%). There was also a strong emphasis on monitoring biochemical indicators (P4&P6) and drug concentrations to guide treatment in sepsis patients (P11) (Table 4).

**Table 4:** Practices.

	Strongly agree	Agree	Neutral	Disagree	Strongly disagree
P1. You often attend academic conferences or training in your relevant field to acquire knowledge about the rational use of antibiotics.	78 (39)	71 (35.5)	40 (20)	9 (4.5)	2 (1)
P2. You frequently consult the latest antibiotic treatment guidelines to update your knowledge.	88 (44)	83 (41.5)	24 (12)	3 (1.5)	2 (1)
P3. You regularly communicate with other doctors/pharmacists to share recent experiences with antibiotic treatments (such as participating in case discussions of septic patients during morning rounds).	86 (43)	78 (39)	27 (13.5)	7 (3.5)	2 (1)
P4. You closely monitor liver and kidney indicators in septic patients to adjust the dosage of antibiotics.	117 (58.5)	68 (34)	12 (6)	3 (1.5)	/
P5. You closely monitor antibiotic blood concentration test results in septic patients to adjust the dosage.	89 (44.5)	64 (32)	32 (16)	15 (7.5)	/
P6. You closely monitor microbiological culture results and biochemical indicators like PCT to adjust the treatment plan.	132 (66)	54 (27)	11 (5.5)	3 (1.5)	/
P7. You emphasize the prevention of drug-resistant bacteria during antimicrobial therapy.	117 (58.5)	69 (34.5)	14 (7)	/	/
P8. Throughout the treatment, you closely monitor antibiotic-related adverse reactions in septic patients.	116 (58)	70 (35)	12 (6)	2 (1)	/
P9. During the treatment, you frequently communicate with patients or their family members to explain the necessity and precautions of antibiotic therapy.	99 (49.5)	65 (32.5)	25 (12.5)	10 (5)	1 (0.5)
P10. In sepsis treatment, you prioritize the total duration of antibiotic administration.	95 (47.5)	83 (41.5)	17 (8.5)	4 (2)	1 (0.5)
P11. During sepsis treatment, you can consider individual differences in patients while paying close attention to potential drug interactions, implementing personalized and precise treatment.	109 (54.5)	76 (38)	13 (6.5)	2 (1)	/

Pearson’s analysis was performed to assess the relationship between knowledge, attitudes and practices. It was shown that attitudes and practices were positively correlated (r = 0.5512, *p* < 0.001) (Table 5).

**Table 5:** Correlation analysis.

	Knowledge	Attitude	Practice
Knowledge	1		
Attitude	0.0273 (*p* = 0.7011)	1	
Practice	−0.0355 (*p* = 0.6177)	0.5512 (*p*<0.001)	1

Multivariate logistic regression showed that attitudes (OR = 1.59, 95% CI: 1.34–1.87, *p* < 0.001), work experience of 15 years and above (OR = 7.17, 95% CI: 2.33–22.0, *p* = 0.001) were independently associated with practices (Table 6).

**Table 6:** Univariate and multivariate.

Practice	Univariate Analysis		Multivariate Analysis	
	OR (95%CI)	*p*	OR (95%CI)	*p*
Knowledge	1.01 (0.76, 1.34)	0.939		
Attitudes	1.47 (1.27, 1.70)	0	1.59 (1.34, 1.87)	<0.001
Gender				
Male	Ref.			
Female	0.99 (0.54, 1.82)	0.981		
Age				
35 years and below	Ref.			
36–45 years	0.82 (0.41, 1.64)	0.577		
46 years and above	0.98 (0.40, 2.37)	0.974		
Residential type				
Rural	Ref.			
Urban	0.59 (0.24, 1.47)	0.577		
Marital status				
Unmarried/Divorced/Other	Ref.			
Married	1.14 (0.45, 2.90)	0.974		
Education level				
Associate’s and Bachelor’s	Ref.			
Master’s	1.15 (0.62, 2.15)	0.641		
Doctorate	2.53 (0.59, 10.7)	0.209		
Work experience				
Less than 5 years	Ref.		Ref.	
6–15 years	1.94 (0.83, 4.53)	0.122	2.19 (0.86, 5.58)	0.099
15 years and above	2.58 (1.04, 6.34)	0.039	7.17 (2.33, 22.0)	0.001
Hospital level				
Tertiary hospital	Ref.			
Other types	0.81 (0.42, 1.57)	0.55		
Current position				
Intensive Care or Emergency Department Physician	Ref.			
Infectious Disease Physician	0.9 (0.07, 10.3)	0.933		
Physician in other departments excluding Intensive Care, Emergency, or Infectious Disease	0.52 (0.21, 1.24)	0.142		
Clinical Pharmacist	0.77 (0.37, 1.56)	0.474		
Hospital Pharmacist	0.54 (0.13, 2.11)	0.376		
Professional Title				
Junior	Ref.			
Intermediate	0.69 (0.27, 1.73)	0.436		
Associate	1.23 (0.51, 2.99)	0.637		
Senior	1.1 (0.32, 3.77)	0.88		
Experience with ICU patient management				
Yes	Ref.			
No	0.74 (0.39, 1.38)	0.348		
Whether hospital’s laboratory offer therapeutic drug monitoring (TDM)				
Yes	Ref.			
No	1.27 (0.60, 2.67)	0.526		

The structural equation model was established to further investigate whether physicians’/pharmacists’ knowledge and attitude toward antibiotic use in sepsis affect their practice, whether attitude plays an intermediary role between knowledge and practice, and whether knowledge can directly affect their practice. The hypotheses underlying this study are as follows: (1) knowledge directly affects attitudes, (2) knowledge directly affects practices, and (3) attitudes directly affect practices (24).

**Figure 1:**
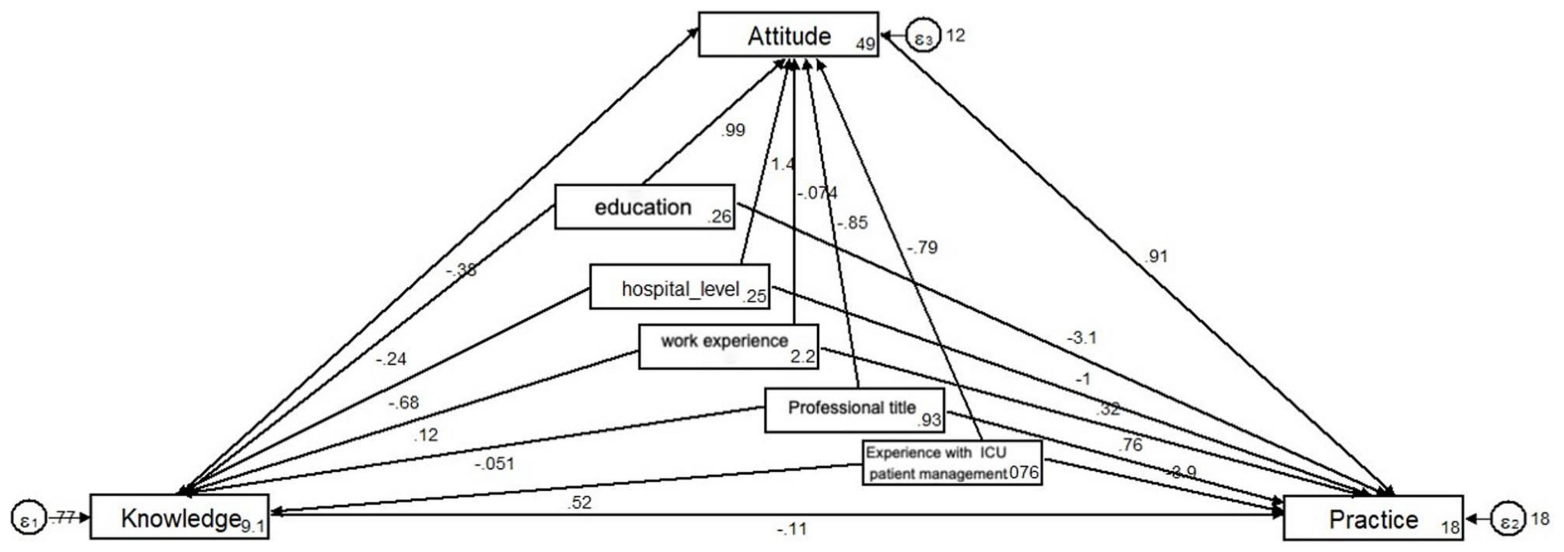
Structural equation modeling (physicians).

For physicians, the fitting index of the structural model (RMSEA = 0.000; SRMR = 0.000; TLI = 1.000; CFI = 1.000) outperformed the respective threshold value, signifying that the data satisfactorily fit the structural model (Table 7). The SEM results support hypothesis (3), showing that attitudes directly affect practices, as indicated by a path coefficient of 0.91 (*p* < 0.001) (Figure 1 and Table 8). However, the SEM did not show a significant direct effect of knowledge on practices, suggesting that while knowledge is essential, its influence on practices may be mediated through attitudes (Figure 1; Table 8).

**Table 7:** Model fitness indices for the KAP structural equation model (physicians).

Indicators	Reference	Results
RMSEA	<0.08 Good	0.000
SRMR	<0.08 Good	0.000
TLI	>0.8 Good	1.000
CFI	>0.8 Good	1.000

**Table 8:** Test results of the hypothesis (physicians).

		Estimate	P > |z|
Knowledge			
	Edu	−0.24	0.284
	Clinical experience	0.52	0.142
	Professional Title	−0.05	0.762
	hospital level	−0.68	0.004
	Years of Work Experience	0.12	0.25
Practice			
	Knowledge	−0.11	0.832
	Attitude	0.91	<0.001
	Edu	−3.07	0.004
	Clinical experience	−3.92	0.022
	Professional Title	0.76	0.346
	hospital level	−1.03	0.396
	Years of Work Experience	0.32	0.532
Attitude			
	Knowledge	−0.38	0.372
	Edu	0.99	0.255
	Clinical experience	−0.79	0.567
	Professional Title	−0.85	0.193
	hospital level	1.38	0.157
	Years of Work Experience	−0.07	0.861

**Figure 2:**
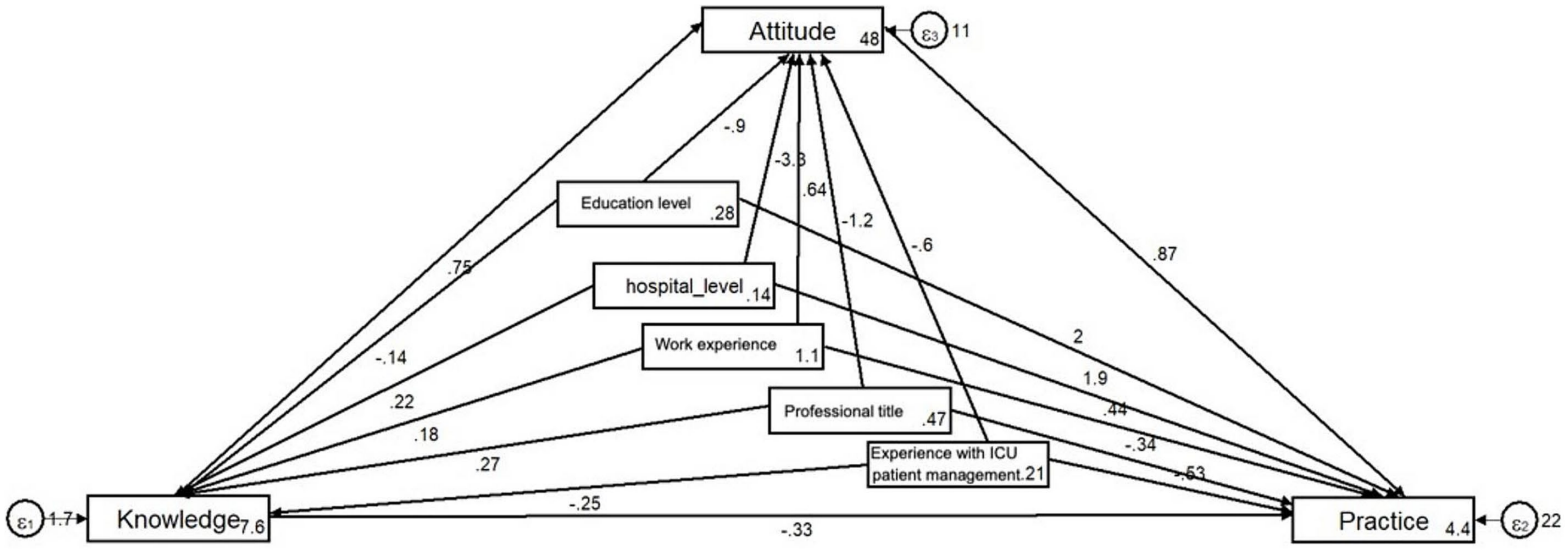
Structural equation modeling (pharmacists).

For pharmacists, the same satisfactory structural model was also fitted (Table 9). SEM showed that knowledge directly affects attitudes, supporting hypothesis (1), with a path coefficient of 0.75 (*p* = 0.024). Moreover, attitudes have a strong direct effect on practices, supporting hypothesis (3), with a path coefficient of 0.87 (*p* < 0.001) (Figure 2; Table 10).

**Table 9:** Model fitness indices for the KAP structural equation model (pharmacists).

Indicators	Reference	Results
RMSEA	<0.08 Good	0.000
SRMR	<0.08 Good	0.000
TLI	>0.8 Good	1.000
CFI	>0.8 Good	1.000

**Table 10:** Test results of the hypothesis (pharmacists).

		Estimate	P > |z|
Knowledge			
	Edu	−0.14	0.684
	Clinical experience	−0.25	0.505
	Professional Title	0.27	0.384
	hospital level	0.22	0.645
	Years of Work Experience	0.18	0.354
Practice			
	Knowledge	−0.33	0.495
	Attitude	0.87	<0.001
	Edu	1.97	0.125
	Clinical experience	−0.53	0.703
	Professional Title	−0.34	0.769
	hospital level	1.90	0.308
	Years of Work Experience	0.44	0.552
Attitude			
	Knowledge	0.75	0.024
	Edu	−0.90	0.318
	Clinical experience	−0.60	0.537
	Professional Title	−1.16	0.151
	hospital level	−3.28	0.009
	Years of Work Experience	0.64	0.215

## Discussion

The findings revealed that physicians and pharmacists have sufficient knowledge, active attitudes, and proactive practices toward antibiotic use in sepsis. Nonetheless, the results also reveal the persistence of certain misconceptions, alongside notable shortcomings in both attitudes and practices, indicating that comprehensive training programs are imperative for enhancing the practices of physicians and pharmacists in this field.

The results of this study indicate a generally positive orientation of physicians and pharmacists toward appropriate antibiotic use in sepsis, as evidenced by high mean scores in knowledge, attitude, and practice. However, certain demographic factors were found to influence these scores, and targeted educational interventions based on professional status and demographic characteristics could address these variations (25, 26).

The results of the knowledge assessment revealed a generally high correct rate among participants, indicating a solid understanding of key concepts related to sepsis and antibiotic management. However, certain deficiencies were identified, particularly in the recognition of distinctions between sepsis and septic shock. A notable proportion of respondents incorrectly asserted that septic shock and sepsis are synonymous, overlooking the crucial distinction of low blood pressure in septic shock. Educational interventions should prioritize emphasizing the unique clinical features of septic shock, ensuring accurate differentiation from sepsis. Additionally, interventions should focus on reinforcing the understanding of prompt antibiotic administration while considering patient-specific conditions, such as renal dysfunction, to avoid suboptimal dosing and potential adverse outcomes (27, 28). These targeted educational efforts can contribute to a more comprehensive and accurate knowledge base among healthcare professionals involved in sepsis management.

The assessment of attitudes toward sepsis management and antibiotic use revealed generally positive inclinations among healthcare professionals. However, certain deficiencies were identified, particularly in the prioritization of branded antibiotics over generics in the event of a significant worsening of a sepsis patient’s condition. A substantial percentage of respondents expressed a preference for branded antibiotics, citing perceived better efficacy and safety. This finding suggests a potential inclination toward a preference for brand names that may not always align with evidence-based practices or cost-effectiveness considerations. Previous studies have highlighted the importance of promoting rational antibiotic use, considering both efficacy and cost-effectiveness, to address concerns related to antibiotic resistance and healthcare costs (29). To improve clinical practice, interventions should focus on antibiotic stewardship principles, emphasizing evidence-based decision-making and cost-effectiveness without compromising patient safety. Additionally, collaborative efforts between healthcare professionals, clinical pharmacists, and pharmacy departments, as expressed in the positive attitude toward collaboration, can be leveraged to enhance antibiotic management strategies. This underscores the need for multifaceted interventions that address knowledge gaps and promote a balanced approach to antibiotic selection in sepsis management.

The evaluation of clinical practices related to antibiotic use in sepsis management indicates a generally positive adherence to evidence-based practices among healthcare professionals. However, certain areas of improvement were identified, such as the prioritization of the total duration of antibiotic administration, which exhibited a lower agreement rate. Previous research has emphasized the importance of individualizing antibiotic duration based on patient response and clinical indicators, rather than adhering strictly to fixed durations, to optimize patient outcomes and minimize the risk of antibiotic resistance (30). To address this deficiency, interventions should focus on reinforcing the principles of antibiotic stewardship, incorporating flexible duration strategies that align with patient-specific factors. Additionally, the practices of monitoring antibiotic-related adverse reactions, communicating with patients or their family members, and considering individual differences should be further promoted (31). These recommendations underscore the need for continuous education and awareness programs to further optimize clinical practices in antibiotic use during sepsis treatment.

One limitation of this study is that the study relies on self-reported data from physicians and pharmacists, which could introduce response bias or social desirability bias. The cross-sectional design provides a snapshot of the participants’ knowledge, attitudes, and practices at a specific point in time, but it does not allow for the examination of causality or changes over time. The use of a web-based questionnaire may also introduce a selection bias, as participants with internet access and familiarity with online surveys may differ from those without. Additionally, as a single-site study conducted within the ICU of Shanxi Bethune Hospital, the findings may be limited in generalizability. Only three infectious disease physicians were included due to the ICU team configuration at this hospital. Thus, they were grouped within the broader physician cohort rather than analyzed separately. Furthermore, significant demographic differences among participants, including age, marital status, work experience, professional title, and ICU management experience, may influence knowledge, attitudes, and practices scores. These discrepancies could limit the conclusions to the major demographic categories represented, and findings should be interpreted with caution when generalizing to broader populations. Despite these limitations, the study offers valuable insights into the current state of knowledge, attitudes, and practices among physicians and pharmacists in the specified setting.

Overall, the results underscore the need for tailored educational interventions that specifically address identified knowledge gaps and misconceptions. Such interventions would promote optimal antibiotic stewardship in sepsis care, improving both clinical decision-making and patient outcomes.

## Data Availability

The original contributions presented in the study are included in the article/Supplementary material, further inquiries can be directed to the corresponding author.

## Footnotes

^1^ http://www.sojump.com
