# Supplementary material for: Knowledge, attitudes, and practices among physicians and pharmacists toward antibiotic use in sepsis
Source: Front Med (Lausanne). 2025 Jan 15;11:1454521. doi: 10.3389/fmed.2024.1454521 (PMC11774854; doi:10.3389/fmed.2024.1454521)
Supplement: Supplementary file 1 [file Table_1.DOCX]

| Questionnaire No. ________________  Dear Doctor/Pharmacist,  Hello!  We are researchers from the _______ Department of _______ Hospital. We sincerely invite you to participate in our study titled "Knowledge, attitudes, and practices among physicians and pharmacists toward antibiotic use in sepsis" The purpose of this study is to provide a scientifically sound tool to evaluate the knowledge, attitudes, and practice (KAP) levels of clinical physicians and pharmacists regarding rational antibiotic use for sepsis, to investigate the current situation, and to analyze the factors affecting their KAP levels. This will offer effective guidance to improve the knowledge base of clinical physicians and pharmacists on rational antibiotic use for sepsis, promote rational antibiotic use in clinical settings, control the risk of bacterial resistance, and improve the prognosis of sepsis patients.  We greatly appreciate your time in filling out this questionnaire, which will take approximately 10-15 minutes. This study will not affect your interests. The data collected will be coded and securely stored, and results will be published in the form of group data. Your name and personal information will not be mentioned, and no information will be disclosed without your permission. Please complete the questionnaire independently without consulting related knowledge. Thank you very much for your support!  □I have been informed and agreed to the use of the collected data for scientific research.  Informed Consent Signature: Telephone Number (optional):  Participation date: Year Month Day | |
| --- | --- |
| **Part I Basic Information** | |
| **1.Your gender:** | a. Male  b. Female |
| **2.Your age:** | a. 18-25 years  b. 26-35 years  c. 36-45 years  d. 46-55 years  e. 56 years and above |
| **3.Your residential type:** | a. Rural  b. Urban |
| **4.Your marital status:** | a. Unmarried  b. Married  c. Divorced  d. Other |
| **5.Your education level:** | a. High school/secondary school and below  b. College and Bachelor's Degree  c. Master's Degree  d. Doctorate |
| **6.What is your work experience in clinical medicine/pharmacy?** | a. Less than 1 years  b. 1-5 years  c. 6-10 years  d. 11-15 years  e. 15 years and above |
| **7.What is the hospital level you work in?** | a. Tertiary class A hospitals  b. Tertiary class B hospitals  c. Secondary class A hospitals  d. Secondary class b hospitals  e. Primary hospitals and other hospitals |
| **8.Your current position is？** | a. Intensive Care Physician  b. Emergency Department Physician  c. Infectious Disease Physician  d. Physician in other departments excluding Intensive Care, Emergency, or Infectious Disease  e. Clinical Pharmacist  f. Hospital Pharmacist |
| **8.1 If you are a hospital pharmacist, what is your professional?** | _________ |
| **9.Your current professional title is？** | a. Junior  b. Intermediate  c. Associate  d. Senior |
| **10.Do you have experience rotating or managing patients in the ICU unit?** | a. Yes  b. No |
| **11.Does your hospital's testing department, pharmacy, or laboratory offer therapeutic drug monitoring (TDM) for antibiotics? (except for tacrolimus, mycophenolic acid, infliximab, and other non-antibiotic drugs).** | a. Yes  b. No |
| **11.1 If you chose ‘Yes’ to Question 11, please tick the type of antibiotic for which TDM is practised in your hospital.** | a. Aminoglycoside antibiotics  b. Penicillin antibiotics  c. Sulfonamide antibiotics  d. Glycopeptide antibiotics  e. Fluoroquinolone antibiotics  f. Other: _______ |

| **Part II Knowledge of clinicians and pharmacists toward rational use of antibiotics in sepsis**   \| **1. Sepsis is a syndrome in which the body's response to infection leads to organ dysfunction, characterized mainly by symptoms such as chills, fever (or low body temperature), palpitations, shortness of breath, and changes in mental status.** \| a. Correct \| b. Wrong \| \| --- \| --- \| --- \| \| **2. Septic shock and sepsis refer to the same disease; there is no distinction.** \| a. Correct \| b. Wrong \| \| **3. Sepsis is solely caused by bacteria; fungi, parasites, and viruses do not cause sepsis.** \| a. Correct \| b. Wrong \| \| **4. Risk factors for sepsis include age (young or elderly), compromised immune system, a history of diabetes or cirrhosis, prolonged intensive care unit stays, trauma, invasive procedures (e.g., intravenous catheters or tracheal intubation), and long-term use of corticosteroids, among others.** \| a. Correct \| b. Wrong \| \| **5. Enterobacteriaceae producing extended-spectrum β-lactamases (ESBL), Pseudomonas aeruginosa, or Acinetobacter species are more sensitive to carbapenem antibiotics.** \| a. Correct \| b. Wrong \| \| **6. Discontinuing antibiotics as soon as clinical judgment determines that a disease is not sepsis, especially when culture results are negative, is an important measure against antibiotic resistance.** \| a. Correct \| b. Wrong \| \| **7. Administer antibiotics promptly after confirming sepsis or septic shock; however, there is no recommendation for a specific target time less than 1 hour.** \| a. Correct \| b. Wrong \| \| **8. Depending on the patient's specific condition, continuous or prolonged administration of β-lactam antibiotics may be considered for sepsis patients.** \| a. Correct \| b. Wrong \| \| **9. When de-escalating treatment, be cautious not to prolong the total duration of antibiotic administration.** \| a. Correct \| b. Wrong \| \| **10. In patients with sepsis and renal dysfunction, adjusting antibiotic dosages may be necessary due to reduced clearance of antibiotics by the kidneys, leading to increased blood drug concentrations.** \| a. Correct \| b. Wrong \| \| **11. Tissue distribution concentrations of antimicrobial drugs are influenced by various factors, including drug properties, tissue types, inflammation severity, blood-brain barrier, placental barrier, and others. Therefore, it is necessary to consider these factors comprehensively when using antimicrobial drugs.** \| a. Correct \| b. Wrong \| \| **12. Septic shock and sepsis are not the same disease; septic shock presents with low blood pressure in addition to the symptoms of sepsis.** \| a. Correct \| b. Wrong \| |
| --- | --- | --- | --- | --- | --- | --- | --- | --- | --- | --- | --- | --- | --- | --- | --- | --- | --- | --- | --- | --- | --- | --- | --- | --- | --- | --- | --- | --- | --- | --- | --- | --- | --- | --- | --- | --- |

| **Part III Attitudes of clinicians and pharmacists toward rational use of antibiotics in sepsis** | | | | | |
| --- | --- | --- | --- | --- | --- |
| **1. You consider timely updating knowledge related to sepsis antibiotic guidelines to be highly important.** | a.Strongly Agree | b.Agree | c.Neutral | d.Disagree | e.Strongly Disagree |
| **2. You are enthusiastic about actively participating in academic conferences related to sepsis and rational antibiotic use, and exchanging clinical experiences about the appropriate use of antibiotics for sepsis with colleagues.** | a.Strongly Agree | b.Agree | c.Neutral | d.Disagree | e.Strongly Disagree |
| **3. As a pharmacist/physician, you are willing to collaborate with clinical departments/pharmacy departments to discuss interventions related to the rational use of antibiotics for sepsis, dose adjustments, blood drug concentration monitoring, and other aspects.** | a.Strongly Agree | b.Agree | c.Neutral | d.Disagree | e.Strongly Disagree |
| **4. During the use of antibiotics, you give importance to patients' biochemical indicators such as PCT, CRP, and microbial culture results to guide the anti-infection treatment plan.** | a.Strongly Agree | b.Agree | c.Neutral | d.Disagree | e.Strongly Disagree |
| **5. You recognize that the overuse of antibiotics can lead to the emergence of drug-resistant bacteria.** | a.Strongly Agree | b.Agree | c.Neutral | d.Disagree | e.Strongly Disagree |
| **6. If antibiotic treatment plans prove to be ineffective, you are willing to proactively communicate with your clinical team and clinical pharmacists to discuss alternative treatment approaches.** | a.Strongly Agree | b.Agree | c.Neutral | d.Disagree | e.Strongly Disagree |
| **7. You maintain a cautious attitude towards off-label drug use.** | a.Strongly Agree | b.Agree | c.Neutral | d.Disagree | e.Strongly Disagree |
| **8. You acknowledge the importance of scientifically implementing de-escalation antibiotic strategies for sepsis.** | a.Strongly Agree | b.Agree | c.Neutral | d.Disagree | e.Strongly Disagree |
| **9. You believe that you have a good grasp of adjusting antibiotic dosages based on liver and kidney function for sepsis patients.** | a.Strongly Agree | b.Agree | c.Neutral | d.Disagree | e.Strongly Disagree |
| **10. You recognize the significance of antibiotic blood concentration monitoring in the treatment of sepsis patients.** | a.Strongly Agree | b.Agree | c.Neutral | d.Disagree | e.Strongly Disagree |
| **11. When the condition of a sepsis patient worsens significantly, do you prioritize the use of branded antibiotics or generic antibiotics?** | a.Branded Antibiotics | b.Generic Antibiotics | c.Both are acceptable |  |  |
| **11.1 If you choose branded or generic antibiotics and not the other, what are your reasons? (Multiple choices):** | a.You believe it offers better efficacy and safety compared to the other formulation. | | b.Considering a cost-effectiveness perspective, you consider it the appropriate choice. | | c.Other: ____ |

| **Part IV Practice of clinicians and pharmacists toward rational use of antibiotics in sepsis** |
| --- |
| 1. **You often attend academic conferences or training in your relevant field to acquire knowledge about the rational use of antibiotics.**   a. Strongly Agree  b. Agree  c. Neutral  d. Disagree  e. Strongly Disagree |
| 1. **You frequently consult the latest antibiotic treatment guidelines to update your knowledge.**   a. Strongly Agree  b. Agree  c. Neutral  d. Disagree  e. Strongly Disagree |
| 1. **You regularly communicate with other doctors/pharmacists to share recent experiences with antibiotic treatments (such as participating in case discussions of septic patients during morning rounds).**   a. Strongly Agree  b. Agree  c. Neutral  d. Disagree  e. Strongly Disagree |
| 1. **You closely monitor liver and kidney indicators in septic patients to adjust the dosage of antibiotics.**   a. Strongly Agree  b. Agree  c. Neutral  d. Disagree  e. Strongly Disagree |
| 1. **You closely monitor antibiotic blood concentration test results in septic patients to adjust the dosage.**   a. Strongly Agree  b. Agree  c. Neutral  d. Disagree |
| 1. **You closely monitor microbiological culture results and biochemical indicators like PCT to adjust the treatment plan.**   a. Strongly Agree  b. Agree  c. Neutral  d. Disagree  e. Strongly Disagree |
| 1. **You emphasize the prevention of drug-resistant bacteria during antimicrobial therapy.**   a. Strongly Agree  b. Agree  c. Neutral  d. Disagree  e. Strongly Disagree |
| 1. **Throughout the treatment, you closely monitor antibiotic-related adverse reactions in septic patients.**   a. Strongly Agree  b. Agree  c. Neutral  d. Disagree  e. Strongly Disagree |
| 1. **During the treatment, you frequently communicate with patients or their family members to explain the necessity and precautions of antibiotic therapy.**   a. Strongly Agree  b. Agree  c. Neutral  d. Disagree  e. Strongly Disagree |
| 1. **In sepsis treatment, you prioritize the total duration of antibiotic administration.**   a. Strongly Agree  b. Agree  c. Neutral  d. Disagree  e. Strongly Disagree |
| 1. **During sepsis treatment, you can consider individual differences in patients while paying close attention to potential drug interactions, implementing personalized and precise treatment.**   a. Strongly Agree  b. Agree  c. Neutral  d. Disagree  e. Strongly Disagree |
